# Three cases of appendicitis with anorexia nervosa under inpatient care

**DOI:** 10.1186/s40337-015-0076-9

**Published:** 2015-11-06

**Authors:** Shu Takakura, Hiroaki Yokoyama, Chie Suzuyama, Keita Tatsushima, Makoto Yamashita, Motoharu Gondou, Chihiro Morita, Tomokazu Hata, Masato Takii, Keisuke Kawai, Nobuyuki Sudo

**Affiliations:** Department of Psychosomatic Medicine, Kyushu University Hospital, 3-1-1 Maidashi, Higashi-ku, Fukuoka-shi, Fukuoka 812-8582 Japan; Department of Psychosomatic Medicine, Graduate School of Medical Sciences, Kyushu University, 3-1-1 Maidashi, Higashi-ku, Fukuoka-shi, Fukuoka 812-8582 Japan

**Keywords:** Appendicitis, Anorexia nervosa, Re-nourishment, Inpatient therapy

## Abstract

**Background:**

Little is known about the occurrence of appendicitis during the re-nourishment period in anorexia nervosa (AN). We report three cases of appendicitis in patients with AN that occurred after hospitalization for treatment of AN.

**Case presentation:**

Case 1 is a 34-year-old female, case 2 is a 17-year-old female and case 3 is a 38-year-old female. Constipation was observed in all three cases. Careful management of defecation might be essential to prevent appendicitis among AN patients during the re-nourishment period under inpatient care. In addition, mild and diffuse symptoms were observed in all three cases. Therefore, diagnosis proved to be difficult to make and abdominal computed tomography was particularly helpful in all cases. As the symptoms were diffuse, the condition of appendicitis turned out to be more severe and complicated in one case. Additionally, the incidence of appendicitis in AN in the current study might be higher than that in the normal population.

**Conclusions:**

These findings suggest that appendicitis should be considered as one of the potentially important complications in the therapy for AN.

## Background

Acute appendicitis is one of the common abdominal diseases and a variety of causes have been reported, including mechanical obstructions caused by fecalith [1], insufficient dietary fiber levels [2], and familial factors [3]. However, there have been no reports concerning the occurrence of appendicitis in patients with anorexia nervosa (AN) during inpatient care.

AN is a chronic and serious disease that presents not only with psychological but also physical symptoms and is accompanied by various physical complications [4–6]. Therefore, careful psychosomatic interventions are mandated [7]. During the re-nourishment period, severe physical complications, such as re-feeding syndrome, have to be taken into account; therefore, treatment strategies for AN need to be considered carefully, such as gradual increase of energy intake [8].

Here, we report three cases of appendicitis in patients with AN during re-nourishment period under inpatient care.

## Case presentation

### Case 1

**Fig. 1:**
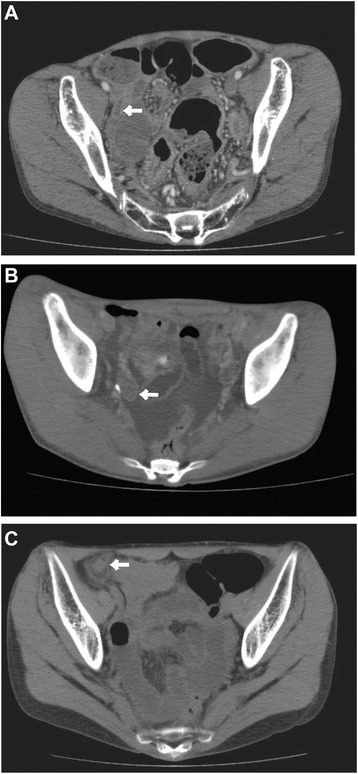
Images of abdominal CT. **a** Case 1, **b** Case 2, **c** Case 3. *Arrow* indicates swollen appendix

This 34-year-old female patient had a fifteen-year history of AN and an eleven-year history of purging behavior such as vomiting after binging and abuse of large amounts of laxative. The patient had regularly consumed 20 tablets/day of irritating laxatives until just before admission to our inpatient treatment unit. The patient’s body mass index (BMI) was 13.6 kg/m^2^. Table shows a summary of the patient’s profile on admission. We started oral energy intake with 1600 kcal/day from day 1 of admission, and the patient was able to consume the prescribed daily dose. We further prescribed a nonirritant laxative at a conventional dose to substitute the irritant laxatives. Although severe constipation improved, mild symptoms remained. On day 24 (BMI 13.6 kg/m^2^), the patient manifested algor and high fever. In the physical examinations, few signs other than very mild abdominal tenderness around McBurney’s point were detected. Leukocytes and C-reactive protein (CRP) values in the blood test were elevated to 21740/μl and 2.48 mg/dl, respectively (Table 1). Abdominal ultrasonography (US) could not detect any abnormality but abdominal computed tomography (CT) showed appendiceal enlargement suggesting acute appendicitis (Fig. 1a). Emergency surgery was performed at the Department of Surgery on the same day. Based on the post-operative pathological examination, the patient was diagnosed with acute phlegmonous appendicitis.

**Table 1:** Summary of patients’ profile and data

	Case 1	Case 2	Case 3	Reference values
Patients’ profile on admission				
Age (year)	34	17	38	-
Diagnosis	AN-BP	AN-R	AN-BP	-
BMI (kg/m^2^)	13.6	12.9	11.9	-
EDI on admission				
Total	101	108	72	-
Drive for thinness	3	19	18	-
Lack of interoceptive awareness	22	16	22	-
Bulimia	13	12	4	-
Body dissatisfaction	11	22	8	-
Ineffectiveness	24	19	6	-
Perfectionism	8	4	9	-
Interpersonal distrust	9	9	5	-
Maturity fears	11	7	0	-
Laboratory data at onset				
White blood cell (/μl)	21400	12890	8240	3500–9000
Neutrophil (%)	89.7	91.5	86.8	40–70
Red blood cell (×10^4^/μl)	406	383	390	385–465
Hemoglobin (g/dl)	12.6	12.2	11.9	12.0–16.0
AST (unit/l)	22	33	38	13–33
ALT (unit/l)	20	79	49	6–30
BUN (mg/dl)	7	12	13	8–22
Cr (mg/dl)	0.67	0.65	0.7	0.40–0.70
CRP (mg/dl)	2.48	1.20	0.09	≧0.10

*BMI* Body mass index, *EDI* eating disorder inventory, *AN-BP* anorexia nervosa binge-eating/purging type, *AN-R* anorexia nervosa restricting type, *AST* Aspartate aminotransferase, *ALT* Alanine aminotransferase, *BUN* blood urea nitrogen, *Cr* creatinine, *CRP* C-reactive protein

### Case 2

This 17-year-old female patient had a 2-year history of AN without purging behaviors. The patient’s BMI was 12.9 kg/m^2^ on admission (Table 1). We started oral energy intake with 1200 kcal/day and intravenous nutrition with 200 kcal/day. We started a nonirritant laxative at a conventional dose because of the patient’s report of constipation. On day 4 (BMI 12.9 kg/m^2^), the patient manifested algor, high fever, and diarrhea. In the physical examinations, very mild abdominal tenderness and increased bowel sound were detected. The leukocyte levels were elevated to 12890/μl and neutrophilia was observed (Table 1). Therefore, we first diagnosed her with acute enterocolitis and immediately started intravenous antibiotics therapy. However, high fever and abdominal pain persisted in spite of the five-day antibiotics therapy. Abdominal US could not detect any abnormality but abdominal CT (Fig. 1b) indicated an intrapelvic abscess on the right side and surgical operation was performed on the next day at the Department of Surgery. The patient was diagnosed with acute gangrenous appendicitis and localized peritonitis intraoperatively.

### Case 3

This 38-year old female patient had an 8-year history of AN with purging behavior such as vomiting after binge eating and laxative abuse. The patient’s BMI was 11.95 kg/m^2^ on admission (Table 1). Pancytopenia was observed in the blood test on admission. We started a nonirritant laxative at a conventional dose because of the patient’s report of constipation after admission; however, transient constipation was observed during the entire inpatient period. The patient’s body weight gradually increased with inpatient therapy. On day 224 (BMI 16.2 kg/m^2^), the patient manifested slight abdominal pain without muscular defense. Pyrexia was not observed and leukocyte values were within normal range (Table 1). CRP level was slightly elevated. Abdominal US could not detect any abnormality but abdominal CT (Fig. 1c) indicated acute appendicitis and surgical operation was performed on the same day at the Department of Surgery. Based on the post-operative pathological examination, the patient was diagnosed with acute phlegmonous appendicitis.

Treatment of AN in all three cases was continued following the successful surgical operations.

In a general year, more than a hundred new patients with eating disorder visit our clinic, and around 30 AN patients are admitted to our inpatient unit. Over a period of recent 3 years, we observed three cases of appendicitis among the inpatients with AN (about 3 %). A report indicated that the lifetime prevalence of appendicitis was 8.6 % in males and 6.7 % in females, and the annual incidence of appendectomy was 11.8 per 10,000 population (0.118 %) in the United States [9]. According to the authors, the highest rate of incidental appendectomy was observed in females aged 35–44 years (43.8 per 10,000 population, 0.438 %), and the rate of appendicitis was found to be higher in Caucasians than in non-Caucasians. Because our study was retrospective and was confined to a small set of cases (i.e., only three) that had been referred to us -rather than having been selected using methods to guarantee statistically representative appendicitis cases- we are cautious about making inferences regarding the incidence or the prevalence of appendicitis associated with AN care. Thus, the incidence of appendicitis during AN treatment in the current study might be higher than that in the general population. Furthermore, although the possibility remains that it was incidental in our cases, appendicitis in all three cases occurred during the re-nourishment period while being hospitalized for therapy. These observations suggest that we should consider appendicitis as one of the gastrointestinal complications of AN under re-nourishment condition.

Gastrointestinal complications, such as constipation, due to chronically abnormal eating behaviors or use of irritant laxatives, are often observed and have been reported among AN patients [10, 11]. However, there have been no reports on the occurrence of appendicitis during the treatment of AN.

Acute appendicitis is one of the common abdominal diseases and a variety of causes have been reported; for example, mechanical obstruction represents one of the causes of appendicitis [1, 12]. Therefore, chronic constipation might be related to occurrence of appendicitis. A tendency for constipation, at varying degrees, was observed in all three cases that showed different AN subtypes. Constipation resulting from inadequate food intake is one of the common complications of AN. Chiarioni reported that most anorexic women complaining of constipation had slow colonic transit times [10]. In addition, among the binge eating/purging type of AN patients, cathartic colon syndrome [13] induced by chronic use of irritant laxatives might be the precipitating cause of constipation. Furthermore, large amounts of stool, observed in the abdominal CT, probably resulted from a combination of adequate food intake and constipation tendency under inpatient condition. To prevent complications of appendicitis, careful control of defecation might be important during the re-nourishment period under inpatient care in AN patients.

The development of appendicitis in Case 3 occurred on the 224^th^ day, while appendicitis development in Case 1 and Case 2 occurred during a relatively early stage of re-nourishment. The underlying cause of this difference in the timing of appendicitis onset is not clear; however, in Case 3, latent constipation may have been a pre-existing chronic condition.

Appropriate diagnosis and treatment is most important to prevent the risk of perforation or peritonitis. None of the three cases manifested severe abdominal symptoms observed in appendicitis such as Rovsing’s sign or Blumberg sign. Especially in case 2, the clinical condition progressed to gangrenous appendicitis and localized peritonitis, as first manifestations had been diffuse and seemed to indicate acute enterocolitis and correct diagnosis was delayed. In case 3, leukocyte level was within normal range, and CRP level was only slightly elevated, probably because pancytopenia had persisted.

To diagnose appendicitis, abdominal US is generally useful and can be the first imaging method of choice [14, 15]. However, in all three cases, abdominal US had difficulty in detecting abnormality. Therefore, we also performed abdominal CT, which proved to be more helpful for diagnosis.

In general, it is more difficult to make a diagnosis of appendicitis if the patient is pregnant [16] or a child [17]. Salo et al. [18] reported that diffuse symptoms in younger children with appendicitis led to delayed diagnosis and complicated appendicitis. Because interoceptive impairments are often reported in the eating disorder inventory [19] among AN patients [20], their perception of bodily signals might be reduced. Thus, similar to the report by Salo et al., mild and diffuse symptoms were observed in the AN cases described above.

## Conclusion

Patients with AN may exhibit diffuse and mild symptoms of appendicitis resulting in difficulties and delays in diagnosis. To prevent appendicitis under re-nourishment during the treatment of AN, careful control of defecation might be important. Though the present study is an observation that there may be a relationship between the cases of appendicitis and AN re-nourishment and is not conclusive proof of cause, these findings suggest that appendicitis should be considered as one of the potentially important complications in the therapy for AN.

## Consent

Oral informed consent was obtained from the patients for publication of this case report and accompanying images.

## Abbreviations

AN: anorexia nervosa
AN-BP: anorexia nervosa, binge eating/purging type
AN-R: anorexia nervosa, restricting type
BMI: body mass index
CRP: C-reactive protein
CT: computed tomography
EDI: eating disorder inventory
US: ultrasonography
